# Quality Indicators for the Pharmacological Management of Chronic Non‐Cancer Pain in Older Adult Patients: An Integrative Review

**DOI:** 10.1111/jep.70253

**Published:** 2025-08-19

**Authors:** Aljoscha Noël Goetschi, Henk Verloo, Nicole Schönenberger, Ursina Wernli, Carla Meyer‐Massetti

**Affiliations:** ^1^ Clinical Pharmacology and Toxicology, Department of General Internal Medicine University Hospital Bern Bern Switzerland; ^2^ Graduate School for Health Sciences University of Bern Bern Switzerland; ^3^ Department of Health University of Applied Sciences and Arts Western Switzerland, HES‐SO Valais‐Wallis Sion Switzerland; ^4^ Institute of Primary Health Care (BIHAM) University of Bern Bern Switzerland

**Keywords:** aged, chronic non‐cancer pain, medication safety, quality indicators, quality of health care

## Abstract

**Rationale:**

Chronic non‐cancer pain (CNCP) affects 28%–88% of older adults. They also experience more medication‐related problems and are more likely to receive insufficient pain therapy, impacting their quality of care. Quality indicators (QIs) can be used to assess and improve the quality of their care.

**Aims and Objectives:**

This integrative review aimed to consolidate the scientific evidence on existing QIs for the pharmacological care of older adult patients with CNCP.

**Methods:**

We systematically searched Medline and Embase via Ovid, CINAHL via EBSCO, the SCOPUS databases and Google Scholar. We used backward citation searching to identify additional studies. We included studies reporting validated QIs and studies reporting quality‐related factors associated with reduced medication safety caused by pain‐related medication therapy. Two reviewers independently screened the titles, abstracts and full texts. One extracted and charted the data and assessed the risk of bias; the second validated this.

**Results:**

We screened 4068 articles identified through the systematic search of databases and 2774 articles identified via citation searching. Seventy‐eight articles met our inclusion criteria and were retained for analysis. We extracted 11 validated QIs and developed a further 243 based on quality criteria reported in the literature. QIs covered different levels, from pharmacotherapy in general to particular substance groups and individual active substances. The risk of bias in the included studies was judged to be very high in the majority of studies.

**Conclusion:**

This integrative review established a scientific basis for developing QIs for the pharmacological management of older adult patients with CNCP. The high risk of bias present in the included studies, highlights the need for expert validation and input from patients. These indicators could help improve the quality of care provided to older adult patients with CNCP and help focus specific interventions on vulnerable patients.

## 1 Introduction

Chronic non‐cancer pain (CNCP) affects 28%–88% of older adults aged 65 or more [1]. These individuals often also suffer from other diseases, the most common being depression and insomnia [2, 3, 4]. Epidemiological data report several factors that correlate with CNCP, including that it affects more women than men [5, 6]. Smoking, alcohol consumption and a poor socioeconomic background, especially past unemployment, are often also observed in combination with CNCP [7, 8, 9, 10, 11]. Lastly, multimorbidity affects up to 88% of people with CNCP [12, 13].

Due to the many factors influencing CNCP, managing it should involve a multimodal approach. Combining medication with physical and psychological therapies may be more beneficial for patients with CNCP than any individual intervention [14]. Interventions that empower patients to self‐manage their chronic pain increase the impact of a coordinated multidisciplinary care plan [14, 15].

Medication for CNCP includes classic analgesics and co‐analgesics. Non‐opioids, such as non‐steroidal anti‐inflammatory drugs (NSAIDs) or paracetamol, and opioids are among those typical classic analgesics. Co‐analgesics are drugs used in pain management for different primary indications. Tricyclic antidepressants and serotonin‐noradrenaline reuptake inhibitors modulate pain perception by increasing noradrenaline concentrations. Sodium or calcium channel inhibitors, such as carbamazepine or gabapentinoids, modulate pain transmission [16, 17].

Older age and CNCP both increase the risk of experiencing medication‐related problems [18]. A medication‐related problem, as defined by the Pharmaceutical Care Network Europe, is an event or circumstance that actually or potentially interferes with desired health outcomes [19]. As such, they include preventable adverse drug events (ADEs) caused by medication errors or non‐preventable ADEs and potential ADEs [20]. Conversely, older adults are also often undertreated for their pain, potentially out of fear of ADEs. This highlights a gap in the quality of care (QoC) for these vulnerable patients [21].

Quality indicators (QIs) have been specifically developed to assess quality [22] and are defined as measurable quantities used to assess care [23]. QIs can be used to track and compare the QoC within and between institutions. When QIs are used as triggers in trigger tools, they enable high‐risk patients or situations to be flagged and allow clinicians to intervene earlier and possibly mitigate these risks [24]. As QIs help standardise health care processes, fairly reward good practices and increase accountability, they have become integral in improving the QoC delivered [23].

The development of QIs requires a rigorous methodology. Campbell et al. suggested that the first step is identifying appropriate QIs through a systematic literature search. The integrative review method achieves this by using a systematic process to critically appraise and synthesise literature. In particular, the integrative review method enables a broader research question to be answered by including different types of study designs (qualitative and quantitative) [25, 26, 27]. The integrative review methodology is therefore optimal for conducting a comprehensive synthesis of all studies reporting on quality criteria within the pharmacological management of CNCP in older adults.

There have been recent efforts to improve the quality of life of older people with CNCP. For example, a Chinese research group developed a set of QIs for community‐dwelling older adults with CNCP. However, the developed set focuses on the overall management process and, therefore, leaves more detailed questions regarding pharmacological management open [28]. In addition, a recent German guideline addresses the management of CNCP in older adults. However, it lacks QIs to measure QoC [29]. As the potential for medication‐related problems and compromised medication safety is higher among older adult patients with CNCP, efforts must be made to improve their QoC. The present integrative review, therefore, aimed to (1) identify studies reporting validated QIs, (2) identify studies from which QIs could be developed, (3) develop new QIs based on these studies, (4) assess the risk of bias in those studies, and (5) propose a first set of QIs that could ultimately be used to enhance older adult patients' QoC.

## 2 Methods

### 2.1 Design

We conducted this review using the Toronto step‐by‐step guide for integrative reviews [27]. Then, we followed the Preferred Reporting Items for Systematic Reviews and Meta‐Analyses Extension for Scoping Reviews (PRISMA‐Scr) for our reporting [30] together with a non‐published protocol available from the authors on request.

### 2.2 Eligibility Criteria

We included studies involving older adult patients aged 65 or older with CNCP. We accepted author‐defined CNCP or pain lasting at least 3 months not caused by cancer. In addition to studies reporting established QIs, we included studies reporting factors associated with reduced medication safety caused by pain‐related medication therapy for older adult patients with CNCP and other articles recommending treatments for them. We included published peer‐reviewed articles in any language and about any type of study and excluded non‐peer‐reviewed articles, conference abstracts, editorials, commentaries and opinion pieces.

### 2.3 Information Sources

We retrieved articles from the Medline and Embase databases via Ovid, from the CINAHL database via EBSCO and from the SCOPUS database, using searches from those databases' inception dates. We also retrieved the first 200 articles from Google Scholar using Publish or Perish software [31] and extracted the articles referenced in them (backward citation searching) using the Citationchaser software [32].

### 2.4 Search Strategy

The search strategy for Medline via Ovid was developed and validated in collaboration with the University of Basel's Medical Library. We then translated the search strategy for Embase via Ovid, CINAHL via EBSCO, and SCOPUS using the automated Systematic Review Accelerator tools [33]. We used a simplified search strategy for Google Scholar, including four thematic blocks covering ‘chronic non‐cancer pain’, ‘medication safety’, ‘quality indicators’ and ‘older people’. Within each thematic block, we combined free text terms and indexing terms (e.g., MeSH and Emtree terms or Subject Headings) using the Boolean operator ‘OR’ and the proximity operator ‘ADJ3’. We combined the four thematic blocks using the Boolean operator ‘AND’. No filters were applied and the search was performed from the respective inception dates. The search string is shown in File S1. The search was carried out on 9 April 2024.

### 2.5 Selecting Sources of Evidence

We extracted articles into EndNote 20.1 software, where we deduplicated them [34]. The articles were then transferred into the Rayyan tool, where the team performed the screening processes [35]. Two reviewers (A.N.G. and C.M.M.) independently screened the titles and abstracts. After retrieving the full texts of the studies selected, two reviewers (A.N.G. and N.S. or C.M.M.) independently made a final decision. In cases of disagreement, the reviewers sought consensus by discussion throughout both processes.

### 2.6 Data Items

We extracted pre‐defined data items as per the review protocol: generic study details and information on the methodology, aims, results and conclusions. We also extracted QIs and their validation status, including information on how they were validated.

### 2.7 Risk of Bias in Individual Studies

One reviewer (A.N.G.) assessed the risk of bias in cross‐sectional, retrospective and prospective cohort studies, mixed‐methods studies, pragmatic, randomised and non‐randomised controlled trials, literature reviews, clinical practice guidelines and qualitative studies. A second reviewer (C.M.M.) validated this. For cross‐sectional studies, we employed the Appraisal tool for Cross‐Sectional Studies (AXIS) [36]. Potential bias in cohort and case‐control studies was assessed using the Newcastle‐Ottawa Quality Assessment Scale (NOS) [37]. Non‐randomised trials, such as pre–post studies, were evaluated using the Risk Of Bias In Non‐randomised Studies—of Exposures (ROBINS‐E) tool [38]. Bias in randomised trials was detected by applying the Cochrane risk of bias 2 (Rob2) tool [39]. Bias in literature reviews was assessed using the Joanna Briggs Institute (JBI) checklist for systematic reviews [40]. The risk of bias in clinical practice guidelines was evaluated using the Appraisal of Guidelines for Research & Evaluation II (AGREE II) instrument [41]. The quality of qualitative studies was assessed using the JBI checklist for qualitative studies [42]. The risk of bias assessment did not influence whether a study was included but was reported alongside the extracted QIs.

### 2.8 Data Synthesis

The mean, median and standard deviation of the age of the overall review population were calculated, together with the gender distribution. All validated QIs were extracted and reported. QIs were considered validated if they were reported as existing instruments and developed using either a systematic literature search (content validity) or a consensus‐building study (face validity) [23]. For studies not reporting validated QIs, new QIs were developed following the Donabedian model [43]. This model differentiates between structural, procedural and outcome‐related QIs. Structural QIs measure static factors that influence the QoC delivered. Examples of structural QIs include the availability of education for health care professionals or the availability of effective treatments. Outcome QIs measure the effects of the QoC delivered to patients. An example could be the number of complications resulting from a treatment. Procedural QIs measure the QoC activities delivered to patients and include, for instance, the drug treatments provided to them. This integrative review focused on developing procedural QIs, which are the most relevant for clinical activities [44].

The data from the newly developed QIs followed an inductive content analysis approach, as the Toronto step‐by‐step guide suggested for conducting integrative reviews [27, 45]. This approach involves first familiarising oneself with the collected studies, then open coding. The codes were then collected in coding sheets and initially loosely grouped. The next step was to reduce the groups by categorising them. Finally, a general description of the categories was formulated through abstraction [45]. This was an iterative process, primarily carried out by ANG. However, the research team regularly discussed the analysis, which provided the primary analyst with new inputs. Due to the abundance of data from which QIs could be formulated, we chose to report only those mentioned three times or more.

## 3 Results

### 3.1 Selection of Sources of Evidence

**Figure 1:**
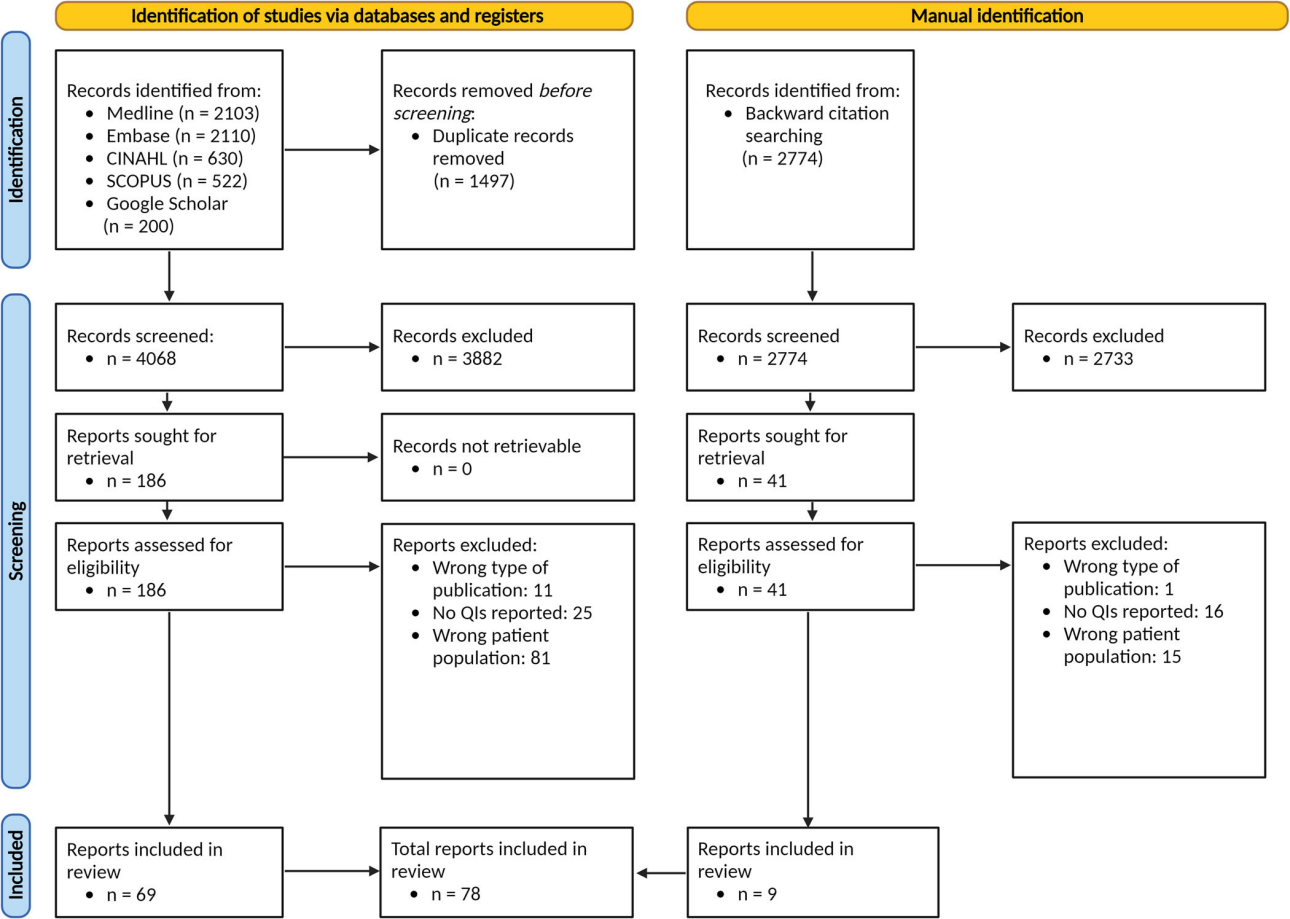
PRISMA flow diagram [30]. Created using biorender.com. QIs = Quality Indicators.

The systematic search across five databases and the backward citation search identified 8339 studies. After deduplication and title and abstract screening, the full texts of 227 studies were assessed, and 78 studies [46, 47, 48, 49, 50, 51, 52, 53, 54, 55, 56, 57, 58, 59, 60, 61, 62, 63, 64, 65, 66, 67, 68, 69, 70, 71, 72, 73, 74, 75, 76, 77, 78, 79, 80, 81, 82, 83, 84, 85, 86, 87, 88, 89, 90, 91, 92, 93, 94, 95, 96, 97, 98, 99, 100, 101, 102, 103, 104, 105, 106, 107, 108, 109, 110, 111, 112, 113, 114, 115, 116, 117, 118, 119, 120, 121, 122, 123] were retained. The PRISMA flow diagram describes the process in Figure 1.

### 3.2 Characteristics of the Sources of Evidence

The studies retained differed in type: most were narrative reviews (*n* = 48, 62%), followed by qualitative studies (9, 12%), cross‐sectional studies (7, 9%), systematic reviews (6, 8%), practice guidelines (4, 5%), case‐control studies (2, 3%), pre–post studies (1, 2%) and mixed methods studies (1, 2%).

Most studies were from the USA (*n* = 45, 58%), followed by Germany (10, 13%), Australia (7, 9%), the UK (3, 4%), the Netherlands (3, 4%), China (2, 3%), Canada (2, 3%), and 1 study (2%) each from Venezuela, Switzerland, Japan, Austria and Italy. One study (2%) was published anonymously.

The overall review population consisted of 25,020 people, of whom 16,206 (65%) were female and 8814 (35%) were male. Studies reported no other or unknown genders. The overall review population's mean age was 71 ± 13 years.

### 3.3 Synthesis of Results

A total of 813 QIs were extracted, although most were not validated. Eight studies (10%) reported validated QIs, of which seven referred to the Assessing Care of Vulnerable Elders (ACOVE) QIs or adapted versions of them, and one referred to the Registry of Senior Australians' (ROSA) QIs. A combined list of these validated QIs is shown in Table 1.

**Table 1:** Summary of validated quality indicators (QIs) with their respective sources. Exact wordings were slightly adapted to fit the scope of this review.

Topic	Indicator	Source
General drug therapy	IF an older adult has a new diagnosis of CNCP, THEN treatment should be provided.	ACOVE, ROSA
General drug therapy	IF an older adult is being treated for CNCP, THEN they should be assessed for a response within 3–6 months.	ACOVE
General drug therapy	IF an older adult with CNCP is new to a primary care practice, THEN there should be documentation of patient education within 6 months explaining the likely cause of symptoms and how to use medication or other therapies.	ACOVE
General drug therapy	IF an older adult with CNCP presents with moderate to severe pain (score ≥ 6 on a scale of 0–10 or a similar quantifiable measure), THEN pain treatment should be adjusted if aligned with care goals.	ACOVE
Opioids	IF an older adult with CNCP is treated using opioids, THEN they should be offered a bowel regimen or medical records should document the potential for constipation or explain why bowel treatment is not needed.	ACOVE
Opioids	IF an older adult with CNCP starts a new opioid therapy, THEN efficacy and side effects should be assessed after between 1 week to 1 month.	ACOVE
Opioids	IF an older adult with CNCP requires analgesia, THEN meperidine should not be used.	ACOVE
NSAIDs	IF an older adult has been prescribed a cyclooxygenase‐nonselective non‐steroidal anti‐inflammatory drug (NSAID) for the treatment of CNCP, THEN medical records should indicate whether they have a history of peptic ulcer disease and, if they do, justification of NSAID use should be documented.	ACOVE
NSAIDs	IF an older adult with CNCP is aged ≥ 75 or has a history of peptic ulcer disease or gastrointestinal bleeding or currently uses coumarin, AND they are treated using a cyclooxygenase‐nonselective NSAID, THEN they should be provided concomitant treatment with misoprostol or a proton pump inhibitor.	ACOVE
Paracetamol	IF oral pharmacological therapy is initiated to treat symptomatic osteoarthritis in an older adult, THEN paracetamol/acetaminophen should be the first drug used.	ACOVE
Paracetamol	IF an older adult's oral pharmacological therapy for symptomatic osteoarthritis is changed from acetaminophen to a different agent, THEN there should be evidence that they have trialled the maximum dose of paracetamol/acetaminophen.	ACOVE

Abbreviations: ACOVE, assessing care of vulnerable elders; CNCP, chronic non‐cancer pain; NSAIDs, non‐steroidal anti‐inflammatory drugs; ROSA, Registry of Senior Australians.

The remaining 70 studies (90%) reported 749 separate QIs, which we amalgamated into 243 QIs. These referred to general drug therapy (37, 15%), opioids (94, 39%), NSAIDs (60, 26%), co‐analgesics (30, 12%) and paracetamol (22, 9%). The median QI was reported once (interquartile [IQR] range = 1–3). To ensure readability, we only report on those QIs mentioned by three studies or more, but all the QIs extracted can be consulted in File S2. Table 2 reports QIs focussing on substance classes, and Table 3 reports QIs focussing on specific drugs. QIs most frequently addressed the need for multimodal, interprofessional treatments, a maximal daily paracetamol dose of 4 g and the monitoring of ADEs under opioid therapy.

**Table 2:** List of quality indicators (QIs) developed categorised into General drug therapy, Opioids, NSAIDs and Co‐analgesics. The number of studies reporting each QI is indicated along with its respective percentage.

Topic		Quality indicator	Studies (%)		Guidelines		Systematic review		Pre–post study		Case control studies		Cross‐sectional studies		Qualitative studies		Narrative reviews
					*n*	RoB (IQR)	*n*	RoB (IQR)	*n*	RoB (IQR)	*n*	RoB (IQR)	*n*	RoB (IQR)	*n*	RoB (IQR)	*n*
General drug therapy		IF an older adult is diagnosed with CNCP, THEN provide multimodal, interprofessional treatment.	25	36%									1	13/18	1	8/10	23
		IF an older adult is diagnosed with CNCP, THEN use base medication combined with as‐needed medication.	5	7%									1	16/18	1	8/10	3
		IF an older adult has CNCP, THEN treat their comorbidities (e.g., insomnia).	3	4%													3
		IF an older adult has CNCP, THEN use the fewest drugs in the smallest doses possible.	3	4%											1	8/10	2
		IF an older adult has CNCP, THEN use more than one drug.	3	4%													3
	Assessment	IF an older adult has CNCP, THEN perform medication reviews regularly.	9	13%	1	59%									1	5/10	7
		IF an older adult has CNCP, THEN monitor for adverse drug events.	6	9%											1	5/10	5
	Galenics	IF an older adult has localised CNCP, THEN use topical drugs.	5	7%			1	5/11									4
		IF an older adult is being treated for CNCP, THEN choose oral drugs.	5	7%													5
		IF an older adult is being treated for CNCP, THEN choose topical drugs.	5	7%	1	60%											4
		IF an older adult has CNCP, THEN use sustained release forms around the clock.	4	6%											1	5/10	3
		IF an older adult has CNCP with continuous pain, THEN use sustained release forms around the clock.	3	4%			1	5/11					1	16/18			1
Opioids	Galenics	IF an older adult with CNCP is treated using opioids, THEN use long‐acting formulations.	11	16%	2	73%	1	4/11									8
		IF an older adult with CNCP is treated using opioids, THEN use short‐acting opioids for breakthrough pain.	9	13%	1	60%			1	*							7
		IF an older adult with CNCP is treated using opioids, THEN use transdermal formulations.	3	4%			1	4/11									2
	Monitoring	IF an older adult with CNCP is treated using opioids, THEN they should be monitored for adverse drug events.	17	24%	3	60% (59‐88%)	1	5/11									13
		IF an older adult with CNCP is treated using opioids, THEN the potential for addiction should be monitored using validated tools.	8	11%	2	59.5%									2	4/10	4
		IF an older adult with CNCP is treated using opioids, THEN their effects should be monitored.	6	9%	1	88%											5
		IF an older adult with CNCP is treated using opioids, THEN renal function should be monitored.	6	9%	1	62%	1	4/11									4
		IF an older adult with CNCP is treated using opioids, THEN hepatic function should be monitored.	3	4%													3
	Dosing	IF an older adult with CNCP is treated using opioids, THEN avoid high doses.	4	6%							1	7/9					3
		IF an older adult with CNCP is treated using opioids, THEN adapt doses to renal function.	3	4%													3
		IF an older adult with CNCP is treated using opioids, THEN adapt doses to hepatic function.	3	4%													3
	Drug–drug Interactions	IF an older adult with CNCP is treated using opioids, THEN other sedative drugs should be avoided.	11	16	1	62%	2	3.5/11							1	3/10	7
		IF an older adult with CNCP is treated using opioids, THEN they should not receive benzodiazepines.	5	7%	1	62%											4
		IF an older adult with CNCP is treated using opioids, THEN fixed combinations with opioid antagonists (e.g., naloxone) should be avoided.	3	4%											3	7/10 (6–7)	0
		IF an older adult with CNCP is treated using tramadol; meperidine or fentanyl, THEN serotonergic drugs should be avoided.	3	4%													3
	Opioids choices	IF an older adult with CNCP has tried all other options without success, THEN consider opioids.	8	11%	1	59%											7
		IF an older adult with CNCP and renal impairment is treated using opioids, THEN use an opioid without active metabolites.	5	7%											1	5/10	4
		IF an older adult with CNCP and hepatic impairment is treated using opioids, THEN use an opioid without active metabolites.	3	4%											1	5/10	2
	Modalities	IF an older adult with CNCP is treated using opioids, THEN use them as part of a multimodal approach.	5	7%	1	88%									1	3/10	3
NSAIDs	General	IF an older adult has CNCP, THEN do not use long‐term NSAIDs.	14	20%	2	59.5%							1	13/18			11
		IF an older adult has CNCP, THEN do not use NSAIDs.	12	17%			1	5/11							4	6.5/10 (3.75–7)	7
	Contraindications	IF an older adult with CNCP has renal impairment, THEN do not use NSAIDs.	15	21%	2	59.5%											13
		IF an older adult with CNCP has heart failure or other cardiovascular diseases, THEN do not use NSAIDs.	13	19%	2	59.5%											11
		IF an older adult with CNCP has peptic ulcers or gastrointestinal bleeding, THEN do not use NSAIDs.	11	16%	2	59.5%							1	13/18			8
		IF an older adult with CNCP has an *H. pylori* infection, THEN do not use NSAIDs.	4	6%	1	60%											3
		IF an older adult with CNCP has hypertension, THEN do not use NSAIDs.	3	4%													3
	Monitoring	IF an older adult with CNCP is treated using NSAIDs, THEN monitor for adverse drug events.	13	19%	2	59.5%	2	4/11									9
	Drug–drug interactions	IF an older adult with CNCP is treated using NSAIDs, THEN do not use multiple NSAIDs.	6	9%	1	60%											5
		IF an older adult with CNCP is treated using NSAIDs, THEN do not combine with corticosteroids.	4	6%	1	59%											3
		IF an older adult with CNCP is treated using NSAIDs, THEN do not combine with ACE‐inhibitors.	4	6%	1	59%											3
		IF an older adult with CNCP is treated using NSAIDs, THEN do not combine with SSRIs.	3	4%	1	59%											2
		IF an older adult with CNCP is treated using NSAIDs, THEN do not combine with diuretics.	3	4%													3
Co‐analgesics	General	IF an older adult has neuropathic CNCP, THEN consider co‐analgesics.	5	7%	1	60%							1	16/20			3
	Antidepressants	IF an older adult with CNCP is treated using TCAs, THEN avoid high doses (10–100 mg)	8	11%	1	60%									3	7/10 (6–7)	4
		IF an older adult has CNCP, THEN avoid TCAs.	7	10%			1	5/11					1	15/20			5
		IF an older adult with CNCP has closed‐angle glaucoma, benign prostate hyperplasia, urinary retention, constipation, cardiovascular diseases or severe hepatic disease, THEN avoid TCAs.	4	6%													4
		IF an older adult has CNCP, THEN do not treat it using SSRIs.	6	9%			1	5/11							3	7/10 (6–7)	2
		IF an older adult has CNCP, THEN do not treat it using SNRIs.	3	4%											3	7/10 (6–7)	0
	Anticonvulsants	IF an older adult has CNCP, THEN do not treat it using gabapentinoids.	3	4%											3	7/10 (6–7)	0
		IF an older adult with CNCP is treated using gabapentinoids, THEN adjust their dose to renal function.	3	4%			1	5/11									2

Abbreviations: ACE‐inhibitors, angiotensin‐converting‐enzyme inhibitors; CNCP, chronic non‐cancer pain; NSAIDs, non‐steroidal anti‐inflammatory drugs; RoB, Risk of bias assessment, where higher numbers or percentages indicate a smaller risk of bias; SNRIs, serotonin‐noradrenalin reuptake inhibitors; SSRIs, selective serotonin reuptake inhibitors; TCAs, tricyclic antidepressants.

**Table 3:** List of quality indicators (QIs) with at least three mentions and addressing individual drugs. The number of studies reporting each QI is indicated along with its respective percentage.

Topic		Quality indicator	Studies (%)		Guidelines		Systematic review		Pre–post study		Case control studies		Cross‐sectional studies		Qualitative studies		Narrative reviews
					*n*	RoB (IQR)	*n*	RoB (IQR)	*n*	RoB (IQR)	*n*	RoB (IQR)	*n*	RoB (IQR)	*n*	RoB (IQR)	*n*
Opioids	Pethidine	IF an older adult with CNCP requires opioids, THEN do not use pethidine.	13	19%			1	3/11					1	13/18	1	7/10	10
	Tramadol	IF an older adult with CNCP requires opioids, THEN do not use tramadol.	6	9%											3	7/10 (6–7)	3
	Pentazocine	IF an older adult with CNCP requires opioids, THEN do not use pentazocine.	4	6%	1	59%							1	13/18			2
	Morphine	IF an older adult with CNCP requires opioids, THEN do not use morphine	4	6%											3	7/10 (6–7)	1
	Fentanyl	IF an opioid‐naïve older adult with CNCP requires an opioid, THEN do not use transdermal fentanyl.	3	4%													3
		IF an older adult with CNCP and renal impairment requires an opioid, THEN use fentanyl.	3	4%													3
	Propoxyphene	IF an older adult with CNCP requires opioids, THEN do not use propoxyphene.	3	4%									1	13/18			2
Paracetamol	Dosing	IF an older adult with CNCP is treated using paracetamol, THEN do not exceed a dose of 4 g per day.	23	33%	2	59.5%	1	5/11									20
		IF an older adult with CNCP and liver disease is treated using paracetamol, THEN adjust the dose.	5	7%	1	60%	1	3/11									3
		IF an older adult with CNCP and chronic alcohol consumption is treated using paracetamol, THEN adjust the dose.	4	6%	1	59%	1	3/11									2
	Contraindications	IF an older adult with CNCP has hepatopathologies, THEN do not use paracetamol.	6	9%	1	60%											5
NSAIDs	Indomethacin	IF an older adult with CNCP requires NSAIDs, THEN do not use indomethacin.	5	7.1	1	59%							1	13/18			3
	Ketorolac	IF an older adult with CNCP requires NSAIDs, THEN do not use ketorolac.	4	5.7	2	59.5%							1	13/18			1
	Naproxen	IF an older adult with CNCP requires NSAIDs, THEN use naproxen.	3	4.3													3
Co‐analgesics	TCAs	IF an older adult with CNCP requires TCAs, THEN use nortriptyline or desipramine.	6	9													6
		IF an older adult with CNCP requires TCAs, THEN do not use amitriptyline.	3	4													3
	Anticonvulsant	IF an older adult with CNCP requires an anticonvulsant, THEN do not use carbamazepine.	5	7											3	7/10 (6–7)	2

Abbreviations: CNCP, chronic non‐cancer pain; NSAIDs, non‐steroidal anti‐inflammatory drugs; RoB, risk of bias assessment, where higher numbers or percentages indicate a lower risk of bias; TCAs, tricyclic antidepressants.

The QIs identified were varied and sometimes contradictory. Differences in QIs could be due to the level of detail. For example, some QIs specified the need to adjust the dose of paracetamol when used in people with liver disease. Other QIs gave specific maximum daily doses. Another discrepancy was in their limitations. While there was a common theme that NSAIDs should be used cautiously, some QIs suggested not using NSAIDs at all, and others suggested not using NSAIDs in subpopulations at high risk (e.g., renal impairment). We found similar patterns for other classes of drugs.

### 3.4 Risk of Bias

The risk of bias was assessed in 31 studies, as shown in detail in File S3. The remaining 47 studies, which were narrative reviews, were not assessed for risk of bias, as we considered this methodology to have an inherent high risk of bias. Cross‐sectional studies scored a median of 16 out of 20 points (IQR = 15–16.75) on the AXIS tool scale. Common limitations of cross‐sectional studies were missing sample size calculation and unclear definitions of the reference population. Both case‐control studies scored 7 out of 9 on the NOS. Neither had independent case validation and exposure was measured only through medical reports. Systematic reviews scored a median of 4.5 out of 11 (IQR = 2.5–6.5) on the JBI tool. Major concerns included missing methodological information and inadequate search strategies. Practice guidelines scored a median of 61% (IQR = 59%–82%) and qualitative studies scored a median of 6.5 out of 10 (IQR = 4.5–7.25) on the JBI tool. In qualitative studies, concerns were often raised about lack of methodological information, panel diversity and auto‐reflexivity.

## 4 Discussion

The present review identified 78 relevant studies and 11 validated QIs. We developed a further 243 QIs from the information extracted, which we categorised under ‘General drug therapy’, ‘Opioids’, ‘NSAIDs’, ‘Co‐analgesics’ and individual drugs (e.g., paracetamol). The QIs developed were mentioned in the literature a median of once. The 70 QIs mentioned in our results were identified at least three times in the articles retained. Most of the studies included were narrative reviews, which we considered to have an inherent high risk of bias.

One significant finding was the variability in the frequency with which each QI was mentioned across the studies. We only identified 11 validated QIs, indicating a paucity of evidence‐based treatment recommendations for older adults with CNCP. In addition, the median QI developed was only mentioned once (IQR = 1–3), with only 70 QIs mentioned three times or more. This variability suggests a lack of consensus regarding the best practices for the pharmacological management of CNCP among older adult patients. The high prevalence of narrative reviews among the studies retained for analysis also suggested a reliance on expert‐opinion rather than evidence‐based approaches for developing QIs [23]. In addition, there are relatively few recent studies on this review's topic. A recent Delphi study developed a set of QIs for the management of CNCP in community‐dwelling adults. The indicators related to pharmacological management are limited to more general recommendations, as this study focused on the whole process of managing CNCP [28]. In addition, a recent guideline lacked QIs to measure the QoC provided to older patients with CNCP [29]. This suggests a cautious interpretation of the results and highlights the need for further validation and standardisation of these QIs.

Certain QIs, such as setting the maximum dose of paracetamol at 4 g per day, were frequently reported in the studies we reviewed. Although this high frequency of reporting suggests both relevance and accuracy, it does not rule out the recommendation's potential for controversy. For example, one analysis of prescribing among older adult inpatients showed that only 28% received more than 3 g of paracetamol per day, suggesting that clinicians prefer more cautious approaches [124]. Indeed, a US Food and Drug Administration Acetaminophen (paracetamol) Advisory Committee Meeting suggested cautious use of paracetamol and a maximum daily dose of 3250 mg [125]. This example highlights the importance of QIs being not only evidence‐based but also reviewed and endorsed by clinical experts, for example, via a Delphi study. Consequently, future efforts to develop QIs should prioritise an expert consensus to ensure their practicality.

Ultimately, QIs must measure improvements in areas directly relevant to patients. Although the development of QIs requires a rigorous methodology and expert input, their true value lies in their ability to reflect improvements in patient care and outcomes [126]. Ensuring that QIs capture meaningful improvements in patient health and satisfaction will increase their usefulness and acceptability in clinical practice and promote a more patient‐centred approach to health care. Therefore, future research and the development of new QIs should also involve patients to ensure that these measures truly reflect and drive the quality improvements that matter most to them.

The validity and feasibility of the QIs reported here may vary across different clinical settings. For example, primary care health records may contain less detailed patient information than hospital records, which may influence the implementation of QIs. Conversely, patients in nursing homes may have different goals and preferences regarding their CNCP management. As this review aimed to summarise QIs for the pharmacological management of CNCP among older adult patients, irrespective of their treatment setting, these QIs may require setting‐specific input from experts and patients to ensure their validity and feasibility. This further highlights the importance of incorporating feedback from different clinical settings into the implementation of these QIs.

The QIs reported here may have several clinical applications. Primarily, they could be used to assess and track the QoC delivered within or between health care institutions. Additionally, QIs could help identify patients at a high risk of compromised medication safety. When integrated into clinical information systems as electronic algorithms, QIs can be efficient tools for identifying patients who might benefit from quality improvement initiatives [23], such as interprofessional medication reviews or patient education. Therefore, integrating these QIs into clinical practice could support efforts to improve patient safety and QoC.

### 4.1 Strengths and Limitations

The present review had some strengths and limitations. We believe that its core strength was combining an extensive systematic literature search over five databases, including Google Scholar, with an in‐depth manual search. Another strength of this review was the division of QIs into validated and non‐validated QIs. Together with the critical appraisal of the studies retained, this enabled us to make evidence‐based decisions on whether a QI should be implemented. A last strength was that the methodology used in this integrative review could serve as a blueprint for developing other QIs from the literature.

However, there are also some limitations to consider. First, although the literature search was validated in collaboration with librarians, we believe the concept of ‘quality indicators’ is quite weakly defined. This may have influenced the accuracy of the search results and led to a greater risk of missing relevant articles. Moreover, the inclusion of more databases might have led to the identification of more studies. We performed an extensive manual literature search and tried to mitigate this risk as best we could. Second, only one reviewer extracted and mapped the data and assessed the risk of bias, which may have introduced bias. Using a second reviewer to validate that work minimised the introduction of bias. Third, the studies retained came mainly from the political West, which may limit the review's generalisability. Fourth, the QIs developed must now be validated by experts in the field and, ideally, other stakeholders, such as patients, before they are ready to use in clinical practice.

## 5 Conclusion

This integrative review identified 11 validated QIs in the literature and developed a further 243 for the pharmacological management of CNCP among older adult patients. The limited number of existing validated QIs, the heterogeneity of the QIs we developed and the prevalence of low‐quality study designs in the articles retained for our analysis all suggest a weak evidence base. This integrative review was the first step in developing validated QIs to support a more evidence‐based approach to managing CNCP among older adults. Further research should focus on developing expert consensus on each of these QIs and involve patients to ensure they are patient‐relevant. These QIs could form the framework for evidence‐based quality improvement projects, ultimately leading to better pharmacological management of older adults experiencing CNCP.

## Conflicts of Interest

The authors declare no conflicts of interest.

## Supporting information

SupplementaryFile1 Searchstrategy.

SupplementaryFile2 QIs.

Supplementary file 3_ROB.

## Data Availability

Data sharing is not applicable to this article as no new data were created or analysed in this study.
