# Supplementary material for: Quality Indicators for the Pharmacological Management of Chronic Non‐Cancer Pain in Older Adult Patients: An Integrative Review
Source: J Eval Clin Pract. 2025 Aug 19;31(5):e70253. doi: 10.1111/jep.70253 (PMC12365588; doi:10.1111/jep.70253)
Supplement: Supplementary file 1 — SupplementaryFile1 Searchstrategy. [file JEP-31-0-s001.docx]

Search strategy on 9 April 2024

**Medline via Ovid**

| **Thema** | **Search String** | **Resultate** |
| --- | --- | --- |
| 1) Chronic pain | exp chronic pain/ OR ((chronic OR persistent OR relentless OR enduring OR constant OR sustained OR continuous OR lingering OR protracted OR life-long OR continual OR continuing OR recurrent OR recurring) adj3 (pain* OR ache* OR cramp* OR spasm* OR colic* OR sore* OR tender* OR burn* OR arthralgia OR headache* OR earache* or backache* or neckache* OR migraine* OR mastodynia OR allodynia OR neuralgia OR hyperalgesia OR myalgia or fibromyalgia or polymyalgia)).ti,ab. | 127629 |
| 2) Medication safety | exp "Contraindications, Drug"/ or exp "Drug Misuse"/ or exp "Inappropriate Prescribing"/ or exp "Medication Errors"/ or exp "Drug Interactions"/ or exp "Drug-Related Side Effects and Adverse Reactions"/ or exp "Drug Information Services"/ or exp "Medication Therapy Management"/ or exp "Potentially Inappropriate Medication List"/ or exp "Drug Utilization Review"/ or exp "Medication Review"/ or (((medication* OR drug* OR medicine* OR medical OR safe*) ADJ3 (error* OR management OR review* OR reconciliation* OR analys* or analyz* OR interaction* OR contraindication*)) OR ((inappropriate* OR inadequate* OR improper* OR incorrect* OR wrong* OR unsuitabl* OR erroneous* OR appropriate* OR adequate* OR proper* OR correct* OR right* OR suitabl*) ADJ3 (medication* OR drug* OR prescription* OR indication* or medicine* or treatment*)) OR ((adverse OR drug?related OR unwanted OR critical) ADJ3 (event* OR effect* OR incidence* OR reaction*))).ti,ab. | 1254846 |
| 3) QI’s | exp "Quality of Health Care"/ or exp "Quality Indicators, Health Care"/ or exp "Guideline Adherence"/ or exp "Quality Assurance, Health Care"/ or ((quality adj3 (predict* or trigger* or indicat* or prognos* or assurance or assess* or tool* or instrument*)) or ((guideline* or protocol* or procedure* or standard* or best practice* or principle* or rule* or regulation*) adj3 (adher* or concord* or congruen* or comply* or complied or complia* or follow* or respect* or accord* or harmon* or respon*))).ti,ab. | 8599527 |
| 4) Elderly | exp aged/ or  (advanced years or ageing or aging or elder* or frail or geriatr* or gerontology* or later life or old age or oldest old or pensioner* or retired or retiree or senescen* or post-menopausal or postmenopausal or senior* or aged or septuagenarian$ or octogenarian$ or nonagenarian$ or centenarian$ or supercentenarian$ or senium).ti,ab. | 4381973 |
| Search combination | 1 AND 2 AND 3 AND 4 | 2103 |

**Embase via Ovid**

| **Thema** | **Search String** | **Resultate** |
| --- | --- | --- |
| 1) Chronic pain | exp "chronic pain"/ OR ((chronic OR persistent OR relentless OR enduring OR constant OR sustained OR continuous OR lingering OR protracted OR life-long OR continual OR continuing OR recurrent OR recurring ) ADJ3 (pain* OR ache* OR cramp* OR spasm* OR colic* OR sore* OR tender* OR burn* OR arthralgia OR headache* OR earache* OR backache* OR neckache* OR migraine* OR mastodynia OR allodynia OR neuralgia OR hyperalgesia OR myalgia OR fibromyalgia OR polymyalgia )).ti,ab. | 204881 |
| 2) Medication safety | exp drug contraindication/ or exp drug misuse/ or exp prescribing error/ or exp medication error/ or exp drug interaction/ or exp adverse drug reaction/ or exp drug information/ or exp medication therapy management/ or exp potentially inappropriate medication/ or exp drug utilization review/ or (((medication* OR drug* OR medicine* OR medical OR safe* ) ADJ3 (error* OR management OR review* OR reconciliation* OR analys* OR analyz* OR interaction* OR contraindication* )) OR ((inappropriate* OR inadequate* OR improper* OR incorrect* OR wrong* OR unsuitabl* OR erroneous* OR appropriate* OR adequate* OR proper* OR correct* OR right* OR suitabl* ) ADJ3 (medication* OR drug* OR prescription* OR indication* OR medicine* OR treatment* )) OR ((adverse OR drug?related OR unwanted OR critical ) ADJ3 (event* OR effect* OR incidence* OR reaction* ))).ti,ab. | 2297383 |
| 3) QI’s | Exp health care quality/ or ((quality ADJ3 (predict* OR trigger* OR indicat* OR prognos* OR assurance OR assess* OR tool* OR instrument* )) OR ((guideline* OR protocol* OR procedure* OR standard* OR "best practice*" OR principle* OR rule* OR regulation* ) ADJ3 (adher* OR concord* OR congruen* OR comply* OR complied OR complia* OR follow* OR respect* OR accord* OR harmon* OR respon* ))).ti,ab. | 4812558 |
| 4) Elderly | Exp aged/ or ("advanced years" OR ageing OR aging OR elder* OR frail OR geriatr* OR gerontology* OR "later life" OR "old age" OR "oldest old" OR pensioner* OR retired OR retiree OR senescen* OR post-menopausal OR postmenopausal OR senior* OR aged OR septuagenarian$ OR octogenarian$ OR nonagenarian$ OR centenarian$ OR supercentenarian$ OR senium ).ti,ab. | 5016934 |
| Search combination | 1 AND 2 AND 3 AND 4 | 2110 |

**SCOPUS**

| **Thema** | **Search String** | **Resultate** |
| --- | --- | --- |
| 1) Chronic pain | TITLE-ABS-KEY( ( chronic OR persistent OR relentless OR enduring OR constant OR sustained OR continuous OR lingering OR protracted OR life-long OR continual OR continuing OR recurrent OR recurring ) W/3 ( pain* OR ache* OR cramp* OR spasm* OR colic* OR sore* OR tender* OR burn* OR arthralgia OR headache* OR earache* OR backache* OR neckache* OR migraine* OR mastodynia OR allodynia OR neuralgia OR hyperalgesia OR myalgia OR fibromyalgia OR polymyalgia ) ) | 185661 |
| 2) Medication safety | TITLE-ABS-KEY(((medication* OR drug* OR medicine* OR medical OR safe* ) W/3 (error* OR management OR review* OR reconciliation* OR analys* OR analyz* OR interaction* OR contraindication* )) OR ((inappropriate* OR inadequate* OR improper* OR incorrect* OR wrong* OR unsuitabl* OR erroneous* OR appropriate* OR adequate* OR proper* OR correct* OR right* OR suitabl* ) W/3 (medication* OR drug* OR prescription* OR indication* OR medicine* OR treatment* )) OR ((adverse OR drug*related OR unwanted OR critical ) W/3 (event* OR effect* OR incidence* OR reaction* ))) | 2419002 |
| 3) QI’s | TITLE-ABS-KEY((quality W/3 (predict* OR trigger* OR indicat* OR prognos* OR assurance OR assess* OR tool* OR instrument* )) OR ((guideline* OR protocol* OR procedure* OR standard* OR "best practice*" OR principle* OR rule* OR regulation* ) W/3 (adher* OR concord* OR congruen* OR comply* OR complied OR complia* OR follow* OR respect* OR accord* OR harmon* OR respon* ))) | 1293135 |
| 4) Elderly | TITLE-ABS-KEY( "advanced years" OR ageing OR aging OR elder* OR frail OR geriatr* OR gerontology* OR "later life" OR "old age" OR "oldest old" OR pensioner* OR retired OR retiree OR senescen* OR post-menopausal OR postmenopausal OR senior* OR aged OR septuagenarian? OR octogenarian? OR nonagenarian? OR centenarian? OR supercentenarian? OR senium ) | 7656779 |
| Search combination | 1 AND 2 AND 3 AND 4 | 522 |

**CINAHL via EBSCO**

| **Thema** | **Search String** | **Resultate** |
| --- | --- | --- |
| 1) Chronic pain | (MH "Chronic Pain+") OR (((TI chronic OR AB chronic) OR (TI persistent OR AB persistent) OR (TI relentless OR AB relentless) OR (TI enduring OR AB enduring) OR (TI constant OR AB constant) OR (TI sustained OR AB sustained) OR (TI continuous OR AB continuous) OR (TI lingering OR AB lingering) OR (TI protracted OR AB protracted) OR (TI life-long OR AB life-long) OR (TI continual OR AB continual) OR (TI continuing OR AB continuing) OR (TI recurrent OR AB recurrent) OR (TI recurring OR AB recurring)) N3 ((TI pain* OR AB pain*) OR (TI ache* OR AB ache*) OR (TI cramp* OR AB cramp*) OR (TI spasm* OR AB spasm*) OR (TI colic* OR AB colic*) OR (TI sore* OR AB sore*) OR (TI tender* OR AB tender*) OR (TI burn* OR AB burn*) OR (TI arthralgia OR AB arthralgia) OR (TI headache* OR AB headache*) OR (TI earache* OR AB earache*) OR (TI backache* OR AB backache*) OR (TI neckache* OR AB neckache*) OR (TI migraine* OR AB migraine*) OR (TI mastodynia OR AB mastodynia) OR (TI allodynia OR AB allodynia) OR (TI neuralgia OR AB neuralgia) OR (TI hyperalgesia OR AB hyperalgesia) OR (TI myalgia OR AB myalgia) OR (TI fibromyalgia OR AB fibromyalgia) OR (TI polymyalgia OR AB polymyalgia))) | 62952 |
| 2) Medication safety | (MH(“Inappropriate Prescribing+” OR “Medication Errors+” OR “Drug Interactions+” OR “Adverse Drug Event+” OR “Drug Information Services+” OR “Medication Management+” OR “Utilization Review+” OR “Medication Review+”)) OR ((((TI medication* OR AB medication*) OR (TI drug* OR AB drug*) OR (TI medicine* OR AB medicine*) OR (TI medical OR AB medical) OR (TI safe* OR AB safe*)) N3 ((TI error* OR AB error*) OR (TI management OR AB management) OR (TI review* OR AB review*) OR (TI reconciliation* OR AB reconciliation*) OR (TI analys* OR AB analys*) OR (TI analyz* OR AB analyz*) OR (TI interaction* OR AB interaction*) OR (TI contraindication* OR AB contraindication*))) OR (((TI inappropriate* OR AB inappropriate*) OR (TI inadequate* OR AB inadequate*) OR (TI improper* OR AB improper*) OR (TI incorrect* OR AB incorrect*) OR (TI wrong* OR AB wrong*) OR (TI unsuitabl* OR AB unsuitabl*) OR (TI erroneous* OR AB erroneous*) OR (TI appropriate* OR AB appropriate*) OR (TI adequate* OR AB adequate*) OR (TI proper* OR AB proper*) OR (TI correct* OR AB correct*) OR (TI right* OR AB right*) OR (TI suitabl* OR AB suitabl*)) N3 ((TI medication* OR AB medication*) OR (TI drug* OR AB drug*) OR (TI prescription* OR AB prescription*) OR (TI indication* OR AB indication*) OR (TI medicine* OR AB medicine*) OR (TI treatment* OR AB treatment*))) OR (((TI adverse OR AB adverse) OR (TI drug#related OR AB drug#related) OR (TI unwanted OR AB unwanted) OR (TI critical OR AB critical)) N3 ((TI event* OR AB event*) OR (TI effect* OR AB effect*) OR (TI incidence* OR AB incidence*) OR (TI reaction* OR AB reaction*)))) | 320096 |
| 3) QI’s | (MH("Quality of Health Care+" OR “Clinical Indicators+” OR “Guideline Adherence+” OR “Quality Assurance+”)) OR (((TI quality OR AB quality) N3 ((TI predict* OR AB predict*) OR (TI trigger* OR AB trigger*) OR (TI indicat* OR AB indicat*) OR (TI prognos* OR AB prognos*) OR (TI assurance OR AB assurance) OR (TI assess* OR AB assess*) OR (TI tool* OR AB tool*) OR (TI instrument* OR AB instrument*))) OR (((TI guideline* OR AB guideline*) OR (TI protocol* OR AB protocol*) OR (TI procedure* OR AB procedure*) OR (TI standard* OR AB standard*) OR (TI "best practice*" OR AB "best practice*") OR (TI principle* OR AB principle*) OR (TI rule* OR AB rule*) OR (TI regulation* OR AB regulation*)) N3 ((TI adher* OR AB adher*) OR (TI concord* OR AB concord*) OR (TI congruen* OR AB congruen*) OR (TI comply* OR AB comply*) OR (TI complied OR AB complied) OR (TI complia* OR AB complia*) OR (TI follow* OR AB follow*) OR (TI respect* OR AB respect*) OR (TI accord* OR AB accord*) OR (TI harmon* OR AB harmon*) OR (TI respon* OR AB respon*)))) | 1055358 |
| 4) Elderly | (MH aged+) OR  ((TI "advanced years" OR AB "advanced years") OR (TI ageing OR AB ageing) OR (TI aging OR AB aging) OR (TI elder* OR AB elder*) OR (TI frail OR AB frail) OR (TI geriatr* OR AB geriatr*) OR (TI gerontology* OR AB gerontology*) OR (TI "later life" OR AB "later life") OR (TI "old age" OR AB "old age") OR (TI "oldest old" OR AB "oldest old") OR (TI pensioner* OR AB pensioner*) OR (TI retired OR AB retired) OR (TI retiree OR AB retiree) OR (TI senescen* OR AB senescen*) OR (TI post-menopausal OR AB post-menopausal) OR (TI postmenopausal OR AB postmenopausal) OR (TI senior* OR AB senior*) OR (TI aged OR AB aged) OR (TI septuagenarian? OR AB septuagenarian?) OR (TI octogenarian? OR AB octogenarian?) OR (TI nonagenarian? OR AB nonagenarian?) OR (TI centenarian? OR AB centenarian?) OR (TI supercentenarian? OR AB supercentenarian?) OR (TI senium OR AB senium)) | 1228421 |
| Search combination | 1 AND 2 AND 3 AND 4 | 630 |
