# Supplementary material for: Quality Indicators for the Pharmacological Management of Chronic Non‐Cancer Pain in Older Adult Patients: An Integrative Review
Source: J Eval Clin Pract. 2025 Aug 19;31(5):e70253. doi: 10.1111/jep.70253 (PMC12365588; doi:10.1111/jep.70253)
Supplement: Supplementary file 3 — _ROB. [file JEP-31-0-s003.docx]

| **Supplementary file 3.** Overview of the risk of bias assessment of all included studies, listed by study design. For narrative reviews, we did not perform any risk of bias assessment, as we considered them to have a high inherent risk of bias. | | | | | |
| --- | --- | --- | --- | --- | --- |
| **Study** | **Country of Origin** | **Study type** | **RoB Tool** | **Score** | **Concerns** |
| U Richarz et al.¹⁰⁷ | Germany | Cross-sectional study | Axis | 15/20 | Unclear how many doctors were excluded for not delivering regular results. Sample size. |
| AF Leone et al.⁸⁵ | USA | Cross-sectional study | Axis | 13/18 | Limitations not discussed, sample size, non responder-bias. |
| J Park et al.⁹⁸ | USA | Cross-sectional study | Axis | 17/20 | Population not representative of reference population, sample size. |
| R Patel et al.⁹⁹ | USA | Cross-sectional study | Axis | 15/20 | Sample size, non-responder bias, statistical methods. |
| J Chodosh et al.⁵⁸ | USA | Cross-sectional study | Axis | 16/20 | Reference population unclear: home dwelling vs vulnerable elders. |
| J Li et al.⁸⁶ | USA | Cross-sectional study | Axis | 17/20 | Major concern is non-representativeness of population. Reference is general community dwelling adults with dementia. But study population is veterans, mostly men enrolled in an RCT. |
| MP Cadogan et al.⁵⁶ | USA | Cross-sectional study | Axis | 16/20 | Main concern is underlying population unclear, as well as selection procedures. |
| M Salkar et al.¹¹⁰ | USA | Case-control study | NOS | 7/9 | No independent case validation, and exposure only via medical report |
| YJJ Wei et al.¹²¹ | USA | Case-control study | NOS | 7/9 | No independent case validation, and exposure only via medical report |
| L Corrado-McKeon et al.⁶⁰ | USA | Pre-Post study | RBOINS-E | Very high risk of bias | Exposure (teaching) is not measured |
| CK O'Neil et al.⁹⁶ | USA | Systematic review | JBI | 3/11 | Methods in general inadequate: no systematic search, no risk of bias, no systematic extraction. |
| A Abdulla et al.⁴⁶ | UK | Systematic review | JBI | 5/11 | No systematic search, inadequate risk of bias assessment. |
| KJ Joling et al.⁸⁰ | Netherlands | Systematic review | JBI | 8/11 | Too few databases, unlcear data synthesis and no publication bias assessment. |
| GE Caughey et al.⁵⁷ | Australia | Systematic review | JBI | 6/11 | Some methodological information not retrievable. |
| S Etzioni et al.⁶⁶ | USA | Systematic review | JBI | 1/11 | Most methodological information not retrievable. |
| AL van Ojik et al.¹¹⁶ | Netherlands | Systematic review | JBI | 4/11 | Much of the methodological information is not retrievable. |
| P Arnstein et al.⁵⁰ | USA | Guideline | AGREE | 59% | Insufficient stakeholder involvement and rigour of development. |
| MW Kahan et al.⁸¹ | Canada | Guideline | AGREE | 62% | Concerns on applicability of guideline. |
| AGS¹²⁷ | USA | Guideline | AGREE | 60% | Concerns on applicability of guideline. |
| W Häuser et al.⁷⁴ | Germany | Guideline | AGREE | 88% | Minor concerns: MDs only healthcare prof. involved. |
| J Pergolizzi et al.¹⁰³ | USA | Qualitative study | JBI | 5/10 | Consensus achieving is unclear and the results are not presented in a diverse manner but as one single recommendation. This seems unrealistic. |
| E Manias⁸⁸ | Australia | Qualitative study | JBI | 8/10 | No auto-reflexivity. |
| N Steel et al.¹¹⁵ | UK | Qualitative study | JBI | 5/10 | Concerns on methods used, because much is unclear, including the panellists. |
| F Pazan et al.¹⁰² | Geramany | Qualitative study | JBI | 7/10 | No auto-reflexivity and concerns about diversity of panellists. |
| F Pazan et al.¹⁰⁰ | Japan | Qualitative study | JBI | 6/10 | No auto-refelxivity, concerns about panellists and methodology perhaps unsuitable. |
| HG Kress et al.⁸⁴ |  | Qualitative study | JBI | 3/10 | Methods largely unreported. |
| A Moser Mays et al.⁹² | USA | Qualitative study | JBI | 7/10 | No auto-reflexivity and concerns about diversity of panellists. |
| JK Goga et al.⁶⁹ | USA | Qualitative study | JBI | 3/10 | Methods largely unreported. |
| F Pazan et al.¹⁰¹ | Germany | Qualitative study | JBI | 7/10 | Major concern is panel: not representative. |
| E Hutt et al.⁷⁸ | USA | Qualitative study | JBI | 8/10 | No auto-reflexivity. |
| E Hutt et al.⁷⁸ | USA | Cross-sectional study | Axis | 16/20 | No major concerns. No sample size calculation. |
| A Velghe et al.¹¹⁸ | Netherlands | Narrative review | None | Deemed high risk of bias. | |
| AM Jablonski et al.⁷⁹ | USA | Narrative review | None | Deemed high risk of bias. | |
| AM Zachary et al.⁸⁹ | USA | Narrative review | None | Deemed high risk of bias. | |
| Anonymous⁴⁸ | ? | Narrative review | None | Deemed high risk of bias. | |
| AR Huang et al.⁷⁷ | Canada | Narrative review | None | Deemed high risk of bias. | |
| AYL Wong et al.¹²² | China | Narrative review | None | Deemed high risk of bias. | |
| B Vegas¹¹⁷ | Venezuela | Narrative review | None | Deemed high risk of bias. | |
| B Vrooman et al.¹¹⁹ | USA | Narrative review | None | Deemed high risk of bias. | |
| BS Workman¹²³ | Australia | Narrative review | None | Deemed high risk of bias. | |
| BST Marie et al.⁹⁰ | USA | Narrative review | None | Deemed high risk of bias. | |
| C McHenry Martin et al.⁹¹ | USA | Narrative review | None | Deemed high risk of bias. | |
| D Dräger et al.⁶³ | Germany | Narrative review | None | Deemed high risk of bias. | |
| D Schneider et al.¹¹² | Switzerland | Narrative review | None | Deemed high risk of bias. | |
| DM Minner et al.⁹³ | USA | Narrative review | None | Deemed high risk of bias. | |
| DRP Guay⁷¹ | USA | Narrative review | None | Deemed high risk of bias. | |
| F Guerriero⁷² | Italy | Narrative review | None | Deemed high risk of bias. | |
| FM Gloth⁶⁷ | USA | Narrative review | None | Deemed high risk of bias. | |
| G Ruoff¹⁰⁹ | USA | Narrative review | None | Deemed high risk of bias. | |
| H Burkhardt⁵⁵ | Germany | Narrative review | None | Deemed high risk of bias. | |
| Iki Chan et al.⁷⁶ | China | Narrative review | None | Deemed high risk of bias. | |
| JB Barber et al.⁵³ | Australia | Narrative review | None | Deemed high risk of bias. | |
| JD Katz et al.⁸² | USA | Narrative review | None | Deemed high risk of bias. | |
| JG Naples et al.⁹⁴ | USA | Narrative review | None | Deemed high risk of bias. | |
| JW Cooper⁵⁹ | USA | Narrative review | None | Deemed high risk of bias. | |
| K Auret et al.¹²⁸ | Australia | Narrative review | None | Deemed high risk of bias. | |
| K Rajput et al.¹⁰⁵ | USA | Narrative review | None | Deemed high risk of bias. | |
| KM Ebener⁶⁴ | USA | Narrative review | None | Deemed high risk of bias. | |
| LA Shimp¹¹⁴ | USA | Narrative review | None | Deemed high risk of bias. | |
| LS Edelman et al.⁶⁵ | USA | Narrative review | None | Deemed high risk of bias. | |
| M Ahmad et al.⁴⁷ | Australia | Narrative review | None | Deemed high risk of bias. | |
| M Kölzsch et al.⁸³ | Germany | Narrative review | None | Deemed high risk of bias. | |
| M Schuler et al.¹¹³ | Germany | Narrative review | None | Deemed high risk of bias. | |
| MC Reid et al.¹⁰⁶ | USA | Narrative review | None | Deemed high risk of bias. | |
| MD Hix⁷⁵ | USA | Narrative review | None | Deemed high risk of bias. | |
| MJ Desai et al.⁶² | USA | Narrative review | None | Deemed high risk of bias. | |
| ML Schilling¹¹¹ | USA | Narrative review | None | Deemed high risk of bias. | |
| MT Owsiany et al.⁹⁷ | USA | Narrative review | None | Deemed high risk of bias. | |
| N Griessinger et al.⁷⁰ | Germany | Narrative review | None | Deemed high risk of bias. | |
| NS Warner et al.¹²⁰ | USA | Narrative review | None | Deemed high risk of bias. | |
| P Arnstein et al.⁴⁹ | USA | Narrative review | None | Deemed high risk of bias. | |
| P Arnstein et al.⁵¹ | USA | Narrative review | None | Deemed high risk of bias. | |
| P Creamer et al.⁶¹ | UK | Narrative review | None | Deemed high risk of bias. | |
| P Robinson¹⁰⁸ | USA | Narrative review | None | Deemed high risk of bias. | |
| RL Barkin et al.⁵⁴ | USA | Narrative review | None | Deemed high risk of bias. | |
| T Hall⁷³ | Australia | Narrative review | None | Deemed high risk of bias. | |
| T Nikolaus et al.⁹⁵ | Germany | Narrative review | None | Deemed high risk of bias. | |
| UE Makris⁸⁷ | USA | Narrative review | None | Deemed high risk of bias. | |
| VK Podichetty et al.¹⁰⁴ | USA | Narrative review | None | Deemed high risk of bias. | |
